# Sustainability Assessment of the Anthropogenic System in Panama City: Application of Biomimetic Strategies towards Regenerative Cities

**DOI:** 10.3390/biomimetics6040064

**Published:** 2021-11-16

**Authors:** Andrea Quintero, Marichell Zarzavilla, Nathalia Tejedor-Flores, Dafni Mora, Miguel Chen Austin

**Affiliations:** 1 Research Group Energy and Comfort in Bioclimatic Buildings (ECEB), Faculty of Mechanical Engineering, Universidad Tecnológica de Panama, Panama City 0801, Panama; andrea.quintero@utp.ac.pa (A.Q.); marichell.zarzavilla@utp.ac.pa (M.Z.); nathalia.tejedor@utp.ac.pa (N.T.-F.); dafni.mora@utp.ac.pa (D.M.); 2 Centro de Investigaciones Hidráulicas e Hidrotécnicas (CIHH), Panama City 0801, Panama; 3 Centro de Estudios Multidisciplinarios en Ciencias, Ingeniería y Tecnología (CEMCIT-AIP), Panama City 0801, Panama

**Keywords:** biomimicry, regenerative design, urban metabolism, green city, sustainability

## Abstract

To understand the sustainability problem for Panama’s metropolitan area, its urban metabolism was investigated. A way to evaluate its current state was obtained by estimating a sustainable indicator based on the Green City Index. With the abstraction of the identified problems, the biomimetic strategy “problem-based approach” was carried out, where different pinnacles of nature were selected as a reference for the design of regenerative solutions. These were inspired by the understanding of the living world and how to include ecosystems in urban designs. Therefore, a framework was proposed for positive generation and natural solutions in cities to take advantage of the regenerative potential in Panama City. Using ecosystem services, a set of indicators were developed to measure regeneration over the years at the city scale. The results indicate that from the 11 selected pinnacles, 17 solutions inspired in nature were proposed to regenerate cities. Consequently, a SWOT analysis was realized along with a questionnaire by experts from different fields. The findings obtained show that the feasible solutions were: arborization, green facades, solar roofs, e-mobility, green corridors, bicycle lanes, sidewalks, and biofilters. This research represents a step towards creating and developing regenerative cities, thus improving the quality of life of living beings and ecosystems present in society.

## 1. Introduction

The considerable increase in the consumption of fossil fuels and the overexploitation of resources are some of the various points that prove how the causes of climate change are anthropogenic. The most polluting human activities include agriculture, energy transformation, industry, such as manufacturing, transportation, and commercial activities. Cities and urbanizations are among the leading causes for this, considering that they consume 40% of the final energy and are related to 70% of global greenhouse gas emissions [1].

Currently, the urban footprint in Panama City is 23 times larger than it was in 1905, where 65.08% of all Panamanians live in urban areas [2]. The urban footprint in the metropolitan area is growing faster compared to the population residing outside these areas.

During the First World Climate Conference, scientists from different parts of the world agreed that climate change trends were alarming. Since then, similar alarms have been raised through the Rio Summit, the Kyoto Protocol, and the Paris Agreement in 2015. Among the globally agreed goals, there are the Sustainable Development Goals; of which this paper will address: Water and Sanitation (SDG 6), Affordable and Clean Energy (SDG 7), Sustainable Cities and Communities (SDG 11), Climate Action (SDG 13), and Life of Terrestrial Ecosystems (SDG 15).

In addition, it is stated that, with the efforts made so far, the global warming of 2 °C will be exceeded during the 21st century, above the objectives of the Paris Agreement, which established limiting the average temperature increase to 1.5 °C. Although, according to the latest United Nations report, over the next 20 years, the global temperature is expected to reach or exceed the 1.5 °C mark unless rapid and deep actions against climate change are made. It is possible to maintain these figures if nations are told to keep their emissions reduction commitments, which should be in the range of 25 GtCO_2_e and 41 GtCO_2_e by 2030 [3]. If these values are exceeded, by the year 2040, there could be a rise in sea level, affecting coastal cities, such as Panama. For instance, a sector that needs real action in its operating and embedded emissions is the energy sector. According to Panama’s National Energy Secretariat, about 70% of the national consumption is concentrated in the capital city, and the National Energy Plan 2015–2050 also states that by 2050, per capita consumption will increase by 90% [4].

In the search for new sustainability strategies in cities that attack these problems, designers have adopted biological research knowledge. In recent years, biomimicry has become relevant in engineering and architecture, giving way to intelligent, self-sufficient, and distinctive buildings in form, structure, and operation. Author Janine M. Benyus gave biomimicry’s first definition in 1997. She defined it as the art of imitating or drawing inspiration from the forms and processes of nature to solve human problems [5].

In architecture, many buildings have already adopted this approach by addressing formal biomimetic design methods in their envelopes and integrating functional solutions, such as the Eastgate Building in Harare (Zimbabwe), the Taichung Metropolitan Opera House, and the Council House 2 (CH2) in Melbourne, Australia.

There are three main levels in biomimicry: organism, behavior, and ecosystem. The organism level refers to a specific organism, such as a plant or animal, where part of it, or the whole organism can be mimicked. The second level refers to the imitation of behavior, where an aspect of behavior is translated into a broader human design context. Finally, the third level is the imitation of entire ecosystems and their principles for working successfully, along with their actual functions. Within each of the levels, there are five possible dimensions; following theses aspects, the design can be biomimetic in how the organism looks (form), what it is made of (material), how it is made or produced (construction), how it works (process), or what it is capable of doing (function) [6].

On the other hand, many of the biomimetic case studies examined by Pedersen Zari in [7], suggested that ecosystem biomimicry may be the most effective way to respond to climate change and biodiversity loss. Yet, right now, ecosystem biomimicry remains the least explored aspect of this branch. A well-known example of ecosystem biomimicry can be seen in the industrial region of Kalundborg (Denmark), where a model of industrial ecology was used. This industrial park is an enduring collaboration between public and private organizations where participants exchange waste materials, residual energy, heat, and water for mutual benefit [8].

Currently, there are two main approaches used in biomimicry, from the perspective of the design problem:

1. The first approach attempts to identify a human design need or problem and then proceeds to look at nature and investigate how certain organisms and ecosystems resolve conflicts; this is known as a top-down or problem-based approach.
2. The second starts with identifying a particular characteristic, behavior, or function belonging to an organism and ecosystem and then investigates different ways of imitating it and adapting it to new designs and products; this is the bottom-up or solution-based approach [9].

In a survey for the strategies that have been taken in different groups of biomimicry practitioners, five of the most recognized groups were discussed: Biomimicry 3.8, Ask Nature, the Biomimicry Institute, Biomimicry Switzerland, and Biomimicry San Diego, concluding that the majority of the groups specialized in the problem-based approach for biomimetic designs [10].

Besides, there are many projects in the current European Union research framework program for nature-based solutions (NBS), known as HORIZON 2020. Many of them strive to explore how NBS works in different urban contexts with respect to the political, social, cultural, institutional, environmental, and economic context [11].

Moreover, the concept of regeneration is emerging extensively in recent investigations by biomimicry practitioners. Pawlyn and Pedersen Zari explained that regenerative systems examine several relevant contemporary examples, which deal with biomimetic technologies and architectures that help the urban built environment adapt to climate change and be favorable factors for the ecological health of the ecosystems. The regenerative design restores the ability of ecosystems to function optimally through the design and development of the built environment [6,12].

One of the concepts most closely linked to regenerative design, which seeks to approach its real application, is ecosystem services. These services are the benefits that humans obtain, directly or indirectly, from ecosystems that support human physical, psychological and economic well-being [6]. The services that humans obtain from the ecosystem are usually divided into provisioning services, such as food and medicine; regulatory services, such as pollination and climate regulation; supporting services, such as soil formation and solar energy fixation, and cultural services, such as artistic inspiration and entertainment. Based on these, strategies have been developed to apply these services in the urban environment [13,14] so that humans themselves contribute to their wellbeing and to the ecosystems with which they coexist. These services apply to the urban developments and can be described as follows:

- Nutrient Cycle: This can be added to cities through food and material imports and lost through exports. It attacks the inability to recover and reuse materials through processes, such as dumping and sewage being discharged to oceans or other regions.
- Habitat Provision: Allows shelter and protection of organisms, providing access to nutritional needs. Such needs are relevant for both permanent and transient populations of organisms and are extremely important for maintaining biodiversity.
- Climate regulation: Regulates processes related to the chemical composition of the atmosphere, the greenhouse effect, the ozone layer, precipitation, air quality and temperature moderation and weather patterns. On a global scale, it encompasses the capacity of ecosystems to emit and absorb carbon and other compounds. In contrast, on a local scale, it considers vegetation to reduce temperatures in urban environments and remove pollutants from the air.
- Purification: Encompasses systems that keep air, water, and soil pure. Urban vegetation is an effective way to remove certain air pollutants, but it is not the only way. Some building materials and filtration systems, for example, can do a similar job and may be more suitable for integration into some types of construction, particularly in medium or high-density areas. Examples are porous metal-organic frame materials, titanium dioxide materials, air ionizers, particulate absorption filters, and other materials.
- Water supply: Includes the regulation of hydrological flows, as well as storage, purification, and water retention. As water is used for consumption for human and animal needs, it is used in large quantities for crop irrigation or other agricultural purposes and industrial processes. Some aspects directly related to the water supply service are water retention, volume management, the timing of runoff, flood control, and drinking water quality.
- Energy provision: The use of biomass and renewable energy is essential as an ecosystem service. Knowledge of energy use will serve as feedback, as an analysis of human behavior and the degradation it causes in ecosystems. However, attempting to replace lost ecosystem services artificially will increase energy, thus leading to further degradation of ecosystems [6].

Examples of ecosystem services analysis (ESA) applied to design include the Lavasa Hill project in Maharashtra, India, and the Lloyd Crossing project proposed for Portland, Oregon. Lavasa was redesigned using the Ecological Performance Standards framework designed by the Biomimicry 3.8 organization and identified six ecosystem services essential to the ecological functioning of the site that was relevant to the development of the urban project in the area. These are water uptake, solar gain; carbon sequestration; water filtration; evapotranspiration, and nitrogen and phosphorus cycling [13].

Considering all the above, the general objective of this study is to conceptualize a reference framework for the proposal of a design methodology based on biomimicry with a vision towards restoration-regeneration at the city scale. This would be done by consulting nature’s models to solve several aspects that must be covered in cities with wrongdoings. The solutions will mainly focus on clean energies, energy efficiency, air purification, urbanism, and sustainable mobility with nature’s strategies and guides. Through this approach, possible opportunities in applying renewal and regeneration in cities are evaluated, using a qualitative and quantitative study through sustainable indicators.

## 2. Materials and Methods

**Figure 1:**
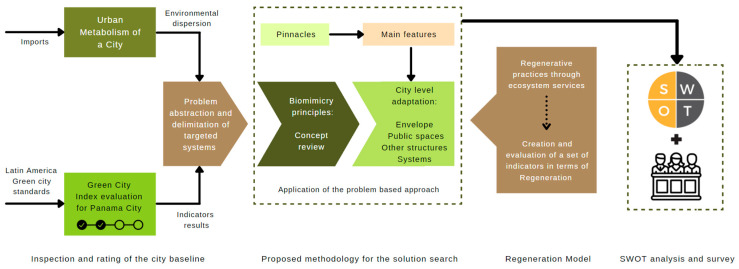
The schematization of the structure proposed for this paper. Own elaboration.

The design of this research was based on the use of a biomimicry problem-based approach to conceptualize and propose a reference framework towards a regeneration model at the city scale. This starts by assessing the current potential for sustainability to identify the main problems within the different systems that make up cities. Figure 1 describes the proposed structure to implement this methodology and the stages that make up this work.

### 2.1. Baseline: Urban Metabolism of the City

The metropolitan area of Panama is governed by a tropical climate, specifically under two main climatic regions: the Central Region (or R4) and the Eastern Pacific Region (or R5). Both have similar precipitation levels, with rainfall decreasing considerably from December to April (dry season) [14]. As a result, Panama’s average outdoor air temperature remains in the range of 23 and 27 °C for the coastal areas and the countryside.

For the temperature of the city itself, the analysis carried out in [15] showed that the area adjacent to Calidonia and Santa Ana had the highest temperatures, ranging from 28 °C to 30.5 °C; while the area with the lowest temperatures was the segment between Clayton and Metropolitan Park with 25.83 °C. This happens since the latter is mostly low-density housing with very dense vegetation. On the other hand, there is a high concentration of economic activity and population in Panama City and the Panama Canal core. Therefore, these territories can be described as “Metropolitan Areas of the Pacific and Atlantic” where approximately 80% of the country’s Gross Domestic Product (GDP) is generated [2].

When it comes to the operation of cities, it can be said that a city works similarly to a superorganism that runs through mechanisms and interactions both internally and with the ecosystem. A similar concept is known as urban metabolism (UM), this can be described as the process in which a city obtains its resources from the local environment or by exchanges, then the city consumes these inputs to produce economic outputs in the form of products and services, and then it releases the residues into the environment [16]. There are different types of UM, however, this paper will be focused on the metabolism that occurs in cities through their carbon footprint.

Due to the urban footprint mostly distributed in the Pacific Metropolitan Area, it was necessary to obtain a delimitation of which systems maintained a larger carbon footprint within its limits or boundaries. Therefore, for this baseline case, the Green House Gas (GHG) emissions figures for each sector were collected.

In the results of the GHG emissions inventory according to the year 2013, the latest updated, each inhabitant of the city’s urban area emitted around 4.90 tCO_2_e. When discussing total emissions, it is denoted that the sector that contributes with the highest emissions is the transport sector, accounting for 46%; this means that the transport system should be taken as a priority. This is followed by stationary sources (residential, services, institutional and industry), which account for 37% of total emissions. The next sector is waste management, with 7%, and the industrial processes and product use (IPPU) sector with 6% of the total. Finally, the energy, agriculture, livestock, and fisheries (AFOLU) sector is a net absorber of CO_2_, with 960,270 tCO_2_e in 2013 [17].

### 2.2. Sustainability Assessment and Problem Definition

There are different ways to evaluate the performance of a city. For instance, among the different urban sustainability indicators, we can mention the Wellbeing Index, which has not been widely used during the last decade due to the appearance of several new sustainability indicators. Besides, the City Development Index (CDI) is considered as a way of measuring urban development and the accessibility to urban facilities. In this regard, between 1993 and 1998, the United Nations Human Settlements Program calculated this index for 232 cities in 113 countries. In conjunction, when evaluating the dimensions measured by the specific index, it is noteworthy that three of them, Ecological Footprint, Environmental Performance Index, and Green City Index, are responsible for covering the environmental and social dimensions. In contrast, the Human Development Index covers the social and economic dimensions [18].

Here, for the case of Panama City, the Green City Index (GCI) is selected, because it has been used since more recent years (2009) and is applicable at the urban scale. Such is the research project conducted by The Economist Intelligence Unit (EIU), sponsored by Siemens, consisting of a series of estimates that began in 2009 and covered more than 120 cities in Europe, Latin America, Asia, North America, and Africa [reference].

Moreover, the GCI has a specific weighting for each indicator; this weighting should be multiplied by the results of the final value for the quantitative data and the qualitative section. Table 1 shows the result obtained from this estimation, where each indicator was compared with a standard value from the guide presented by the Latin American Green City Index [19]. This standard has an optimum minimum and maximum value for a city that is on average sustainable. Data for calculations were obtained from scientific publications, statistics, regulations, and plans or studies generated by the Panamanian government. See Appendix A for the detailed table.

According to the weighting for all indicators in the GCI, the city is within the average sustainable performance in the total result, with 55.95%. However, in the case of the individual indicators, there is an alarming risk in terms of air quality, with 16.67%. For the transportation indicator, there is below-average management, with 39%. On the other hand, the sections on land use, with 47.95%, and energy and CO_2_, with 51.14%, despite being within the average, are at moderate risk and they could be improved to achieve the programmed agenda of goals. Due to this distribution, the priority sectors for action must include air quality and transportation. Additionally, the Energy and CO_2_ sectors and Land Use and Buildings are rated as average with the same need for intervention. These were considered in the analysis due to their contributions to GHG emissions, inorganic waste, and pollution, which are significantly high.

Some of the indicators used for this index showed a score of 0.00 because of a lack of reliable data sources, as in the sulfur dioxide level indicator. In some cases, values exceeded the internationally established standards (20 μg/m^3^ maximum) [19], for example, in the levels of nitrogen dioxide in Panama (36 μg/m^3^) and suspended particulate matter (49 μg/m^3^).

Other indicators also exceeded the standard, such as length of the collective transportation network (0.03 km/km^2^, which is much lower than the minimum of 0.30 km/km^2^), electricity consumption per capita (2226 kWh/inhab/yr, which is higher than the maximum of 815 kWh/inhab/yr) and water consumption per capita (274 L/inhab/day vs. the maximum of 126.90 lt/inhab/day).

The GCI score for the Pacific Metropolitan Area of Panama (1.5 million inhabitants) was favorable, however, it ranks poorly in transport and air quality compared to other cities in the index with similar populations, such as Quito (2.1 million), Curitiba (1.8 million), Montevideo (2 million) and Porto Alegre (1.4 million). Table 2 presents the comparison of GCI results in these cities.

**Figure 2:**
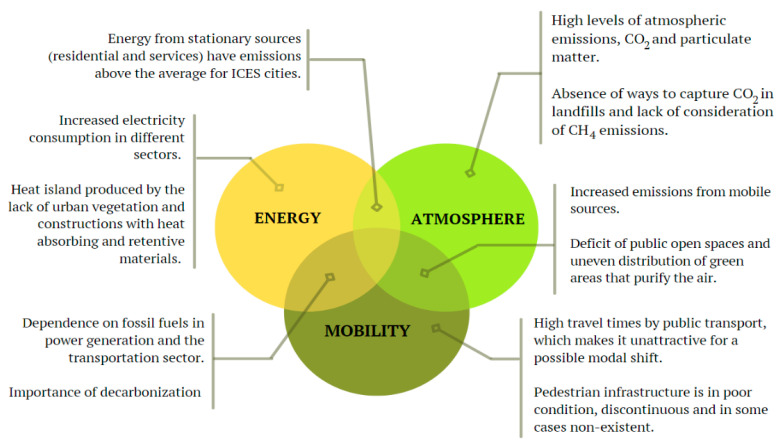
Categorization of the identified problems as a priority. Own elaboration.

A biomimetic analysis of the problem-based approach is sought, where the problems encountered through the index were classified into elements, and those of priority were chosen. In Figure 2, a scheme of the most significant issues among the city sectors is presented to highlight their role in the GHG emissions contribution.

According to these problems, some of the challenges defined to start searching in nature include the following:

- Reducing the energy loss for air conditioning of spaces and increasing cooling efficiency by dissipating excess heat. This leads to identifying “heat regulation” as a challenge.
- Finding better ways to “produce energy” without generating emissions, focusing on organisms in nature that take advantage of solar radiation.
- Determine how nature performs its atmospheric decontamination; how it traps particles or reduces carbon in the air. Thus, this challenge will focus on purification and filtration.
- Exploring alternatives to the motorized mobility for reducing its emissions, focusing on the strategies of nature’s organisms to “transport” themselves while foraging and communicating with each other.

### 2.3. Biomimicry Abstraction: Search for Biological Analogies

Since the most affected sectors were identified (Table 1) as the ones related to the GHG emissions, along with the challenges involved, the search for biological analogies is now performed to accomplish successful biomimicry abstraction. For this, the main problem of reducing such GHG emissions was examined from the point of view of three main elements: energy, mobility, and atmosphere. These will be the themes to be followed during the search for biomimetic solutions. For each topic, a study of the most essential processes that nature performs on its own is presented.

A method of exploration based on the one presented by [20] known as “BioGen” was included in this paper. Developing a biomimetic design solution, required executing multiple stages, such as the following:

1. To elaborate and analyze the pinnacle’s strategies and principles.
2. Analyzing, classifying, and abstracting those strategies.
3. To combine the different strategies options and seek to integrate them to make a preliminary design concept.
4. To evaluate and verify solutions in that design.

The pinnacle search required classifying the different strategies used by living organisms. This was possible by a comprehensive biological literature review. Some of the sources used were Ask Nature, Biomimicry 3.8, and expert knowledge from different sectors.

**Figure 3:**
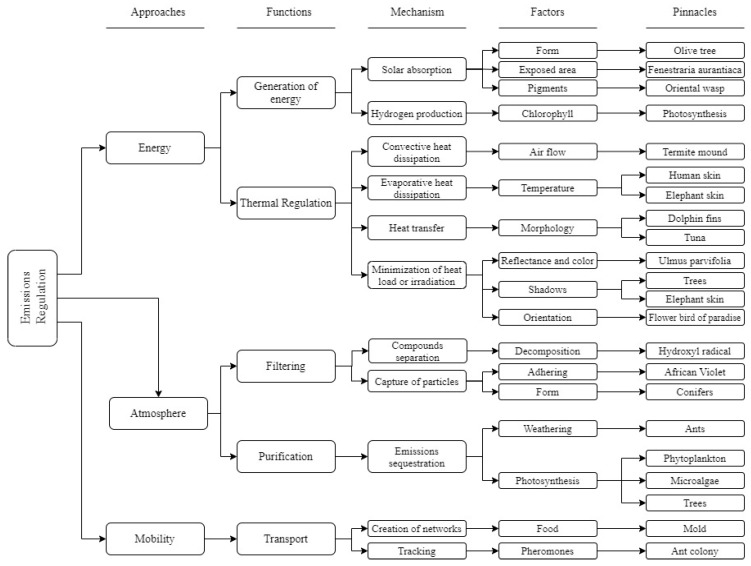
Exploration model for energy, atmosphere, and mobility approaches. Own elaboration.

Figure 3 presents the exploration model intended to be covered. The three elements or approaches (energy, atmosphere, and mobility) were segregated into four levels of exploration. These levels are the function level (the challenges to be explored, what they need to do), mechanism level (how they handle the identified function), factor level (they affect the distinguished processes or are ways of performing that mechanism), and finally, the pinnacle level, representing the example of nature that complies with the previous levels for that function.

For selecting essential pinnacles, first, the main forms of energy production were considered, by selecting those pinnacles that perform processes using the sun and water, which could be replicated in cities, taking plants and *Fenestraria aurantiaca* as examples.

In thermal regulation, pinnacles that could reduce the environment’s temperature, either by evaporation or convection, were extracted, e.g., how the elephant’s skin works with evaporative cooling and the termite mound’s natural ventilation. Finally, analogies were sought in nature that minimizes solar irradiation, with a particular focus on shading; hence, the role of trees and the orientation of the flower *Strelitzia*, were considered.

In filtering, attention was paid to examples from nature with surfaces where particles adhere, as does the *Saintpaulia*. This was taken as a priority rather than other features mentioned in the model, such as the aspect “shape”.

Purification was more linked to the sequestration of CO_2_, VOCs, and other harmful substances, where the biological analogy of microalgae was selected to add to the design’s features. In addition, the use of trees as natural air purifiers was considered because of the amount of pollutants they absorb, while also providing shade for the heat regulation function.

Finally, strategies focused on route optimization were chosen in the transportation challenges, such as ant colonies and the *Physarum polycephalum* mold. Table 3 shows the strategies carried out by each selected pinnacle for this model.

To reduce the search and complexity of the different pinnacles, an imaginary pinnacle is evaluated for each category analyzed. The “X” symbols will denote the corresponding characteristic for each pinnacle according to the category analyzed. Then, the imaginary pinnacle will acquire the most dominant characteristic in every category, which is repeated in two or more pinnacles. If there are no coincidences, all features will be inherited by the imaginary pinnacle.

**Figure 4:**
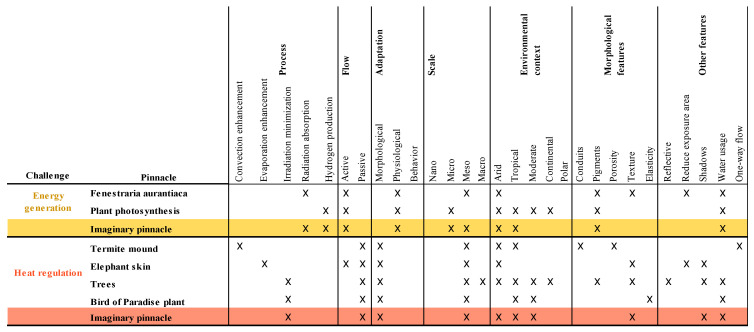
Pinnacle analysis matrix for the energy approach. Own elaboration.

The analysis matrix of the pinnacles selected for the energy approach is presented in Figure 4. The relevant characteristics for power generation and heat regulation are highlighted in the yellow and red box, respectively, representing the imaginary pinnacle for each challenge to be analyzed.

**Figure 5:**
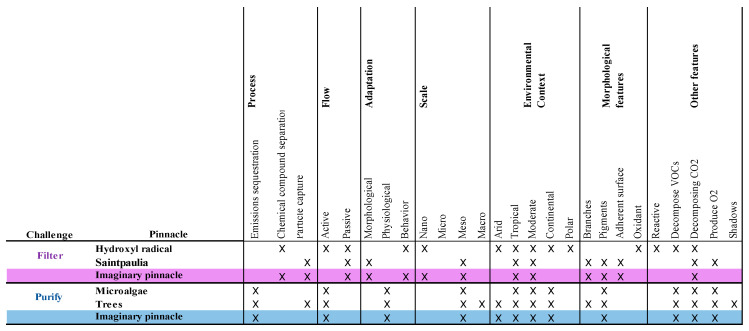
Pinnacle analysis matrix for the atmosphere approach. Own elaboration.

The analysis matrix for the atmosphere approach and the selected pinnacles are presented in Figure 5. Features relevant to filtration and purification are highlighted in the purple and blue box, respectively, representing the imaginary pinnacle of each challenge.

**Figure 6:**
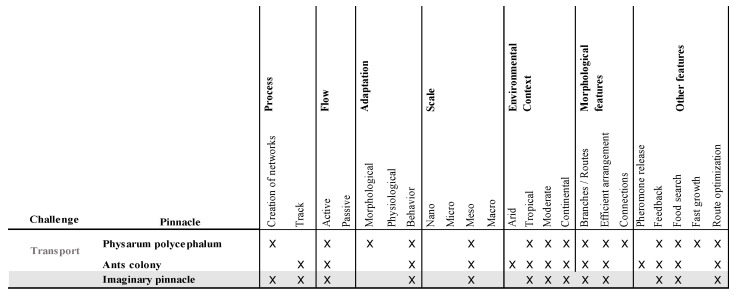
Pinnacle analysis matrix for the mobility approach. Own elaboration.

Finally, Figure 6 shows the analysis matrix with the pinnacles selected for the mobility approach. The relevant function for this challenge was transport, in grey. In this analysis, the chosen pinnacles had behavioral adaptations, and both required constant feedback, allowing them to find optimal routes in their search for food.

### 2.4. Characteristics of the Pinnacles

According to the three design-pathway matrices, they indicated several dominant properties within the different categories relevant to the design concept, these are:

Passive flow predominated in heat regulation and filtration; however, active flow persisted in purification, generation, and transport.

The influence of morphological adaptation was observed in all heat regulation and particle capture processes, while physiological adaptation was more prevalent in energy generation and purification. Finally, for both mobility functions, behavior predominated.

The mesoscale is considered relevant in all the functions presented. However, the environmental context was precise: tropical for each function, and arid, or moderate only in certain processes. Therefore, pinnacles that share the same climate as Panama were considered more relevant.

The morphological characteristics most present in the pinnacles were pigments for energy generation, filtration, and purification, texture, heat regulation, and ramifications in transport. Other characteristics to be considered in the bio-inspired solutions are:

- Solar utilization for energy generation.
- Reduction of the exposed surface and use of shading for heat regulation.
- Reflectance to minimize irradiation.
- Improving adhesion for filtration.
- Compound decomposition and sequestration of CO_2_, VOCs and O_2_ production.
- Feedback and pathways in the design for mobility.

### 2.5. Solutions Based on Nature

In accordance with the most dominant characteristics of the biomimetic abstraction, it is proposed that certain strategies influenced by nature get adopted in Panama City, taking into consideration ideas from the biomimetic model, such as:

- Shading: use of trees, roofs, and louvers.
- Pigments: trees, microalgae, and plants on green roofs and/or walls.
- CO_2_ reduction: filters, vegetation, green hydrogen.
- Solar utilization: photovoltaic panels and solar sheets.
- Routes or branches: sidewalks and green corridors.
- Morphology: applied to buildings, louvers, and sidewalks.
- Passive behavior: found in buildings, roofs, bus stops, and sidewalks.
- Dynamic behavior: found in green roofs, green corridors, microalgae filters, emissions sequestration, non-motorized mobility, and electric mobility.

The dominant characteristics presented from the selected pinnacles will be part of a proposal for regenerative solutions in Panama City. Table 4 presents a description of these proposals and their successful applications.

## 3. Results Analysis

Regenerative urbanization seeks to develop a built environment that coexists with ecosystems and enhances their health instead of diminishing it. However, ecosystems’ services to society are currently not adequately protected because of cities’ poor regulation, solutions, and policies. Therefore, urbanization must contribute more than it consumes to ecosystems, while also remedying past and current actions in terms of environmental damage. This would allow moving towards truly regenerative efforts.

Since it is greatly difficult to replace all buildings and infrastructure for regenerative development, even with retrofitting techniques, an alternative would be to provide ecosystem services in humanity’s own way, to reduce the existing pressure on local ecosystems [49].

**Figure 7:**
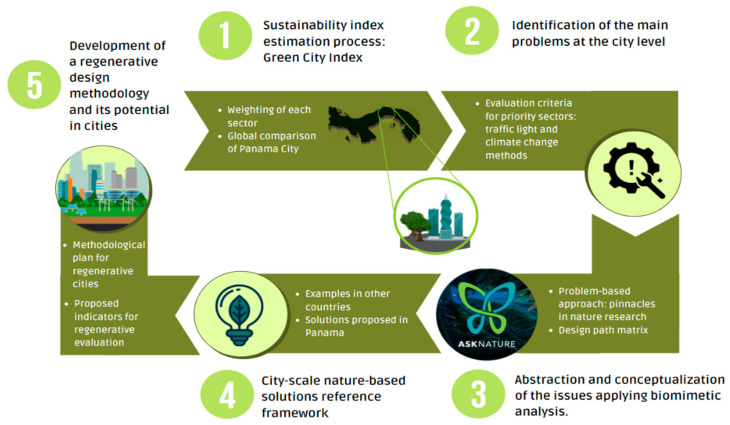
Roadmap for the development of a methodology towards regenerative cities. Own elaboration.

In the previous section, different sustainable alternatives based on nature were discussed to contribute to Panama City’s capacity to be regenerative. As a result, it is possible to respond to the problems arising from poor sustainable management and, in general, to the impacts of climate change, through biomimetic solutions and the so-called ecosystem services analysis (ESA). Figure 7 presents the trajectory of a roadmap proposal for regeneration in cities.

Ecosystem services analysis can work as a starting point for the creation of a regenerative design that is measurable. This is vital to establish the credibility of regeneration in urban design [49]. As a strategy to measure the sustainability of urban regeneration and ensure the principles of sustainable development, a set of indicators will be employed, which focus on the ecosystem services described above. These indicators are described and evaluated in Table 5.

These indicators could be considered for the objectives and targets established in the last Nationally Determined Contribution (CDN1) of Panama, for a circular economy, energy, resilient human settlements, sustainable infrastructure, forests, and biodiversity.

## 4. Discussion

The distribution of the urban footprint, which began to increase with the construction of the Panama Canal, may have affected the city’s position today. Considering other factors that were part of the urban growth, some studies point to the real estate explosion as an important cause, which began in the central banking and financial area of the city (Bella Vista and San Francisco); however, it has been moving to the north and east of the capital city [57]. The existence of multiple, poorly structured zonings in the city, where different land uses are dispersed, is part of the problem. This results in poor connections between areas with only a complicated network of roads, dense streets, deteriorated highways, and unfinished train lines, which increases the travel time of the inhabitants. This is due to the poor distribution of residential areas and economically unbalanced neighborhoods lacking basic services. Some authors and urban planners in [58] define Panama City with the words “half a city”, “a divided city” or “two realities”.

According to INEC data, in the 2010 census, 42% of households in the metropolitan area had a car. However, there were no initiatives to prioritize pedestrians until 2014, when the mayor’s office started interventions for public space. Three restructured projects stand out and serve as an example: Via España, Via Argentina and Calle Uruguay [58]. Because of these and other problems, tools like the Green City Index [19] could estimate a starting point for describing the primary needs in terms of sustainability in cities and urbanizations. These needs can be adapted into biomimicry’s different strategies, to seek better solutions, as has been seen in the literature. Furthermore, nature’s opportunities for urban development offer an efficient way for human advancement in sustainability.

### 4.1. Evaluation of Proposed Approaches via SWOT Analysis

In Section 2, different solutions based on nature were proposed, however, it is not possible to know the suitability of such solutions to an urban and continuously developing environment, such as Panama City. For this reason, it is necessary to discuss or assess the potential for its adaptation; where strengths, weaknesses, opportunities, and threats are sought to be explored through a SWOT analysis for each approach studied.

A strength considers the resources involved in the urban area that make it possible to achieve the objectives considered in the area’s social structure and physical conditions. On the other hand, a weakness focuses on the limitation that prevents the project from achieving the results or objectives in the urban environment. This analysis discusses the initiatives for the energy, air quality, and mobility sectors in Table 6, Table 7, and Table 8, respectively.

### 4.2. Experts Survey for the Evaluation of Regenerative Proposals

As a more exhaustive demonstration of the possibility of adapting the solutions presented for Panama City, a questionnaire was conducted among researchers and experts in different fields involved in the technological and sustainable development of cities. This could be done in collaboration with professionals in energy, environment, architecture, urbanism, among others.

#### 4.2.1. Information Regarding the Participants

In August (2021), the survey was open for responses, and 13 participants gave their opinions through a set of evaluations. It was completed with a relative majority of participants who worked in the public sector, with 69.2% of respondents; the rest, 30.8%, belonged to the independent sector and none to the private sector. The work experience was distributed as follows: 30.8% for more than 20 years, 30.8% for both 10 to 20 years and less than 5 years, and at last, 7.7% for 5 to 10 years.

In addition, as a basis for their knowledge in biomimicry and regeneration, they were asked about their level of understanding, with 61.5% describing themselves as having low knowledge of biomimicry, 23.1% of medium, and only 15.4% having a high level. In the case of regeneration, almost half of them had a medium understanding with 46.2%, 23.1% had insufficient knowledge, 15.4% of them agreed to have high knowledge, 7.7% had a very high understanding, and just 7.7% had very low knowledge. When asked about what topic they considered to have more knowledge in, 76.9% answered in Environment, 23.1% chose Clean Energies, and only 7.7% said Urbanism and Mobility.

#### 4.2.2. Rating Questions and Answers

Local experts were asked which sectors they considered to be priorities for sustainable development in Panama City with the options of 1 (low importance), 3 (neutral), and 5 (high importance). A total of 61.54% considered the waste sector as high importance, making it the most relevant. For the energy and CO_2_ emissions, 53.85% rated it as high importance, and the same happened for land use/buildings, water and sanitation, and transportation sectors. On the other hand, for air quality, only 23.08% considered this sector to be of high importance, 38.46% between very important and neutral, and 38.46% recognized it to be neutral.

Participants were also asked what aspects they considered the most relevant for developing a sustainable city. Their responses pointed to a greater appreciation for the integration of nature and the application of waste reduction measures, both of which were approved by 76.9% of the participants surveyed. This was followed by implementing clean energy with 69.2% and sustainable mobility where the participants indicated their approval with 61.5%. Furthermore, 46.2% valued compact urbanization, while 30.8% valued adequate care of air quality. Finally, a total of 23.1% considered the preservation of public spaces.

To obtain the experts’ opinions for the evaluation of proposed solutions, it was explained what the initiatives consisted of. Then they were asked how they would rate the feasibility of those solutions on a scale from 1 to 5, where 5 denoted high likelihood, 3 meant medium likelihood, and 1 indicated low likelihood.

The results revealed that out of the 17 actions assessed by the respondents, nine of them were considered to have an average value of 4 and upon the scale presented, i.e., close to a high possibility of implementation. The solution that had the highest score for its positive application in cities was the adaptation of trees and plants for arborization. In the case of the remaining eight solutions, they all obtained an average value between 3 (medium) and 4, denoting a medium-high possibility of implementation. No solution had a value lower than 3.31, belonging to the Sierpinski ceiling. These results can be further seen in Table 9, along with the rank occupied by each solution, where rank 1 represents the best-voted option for adaptation, and the value 12 is the least voted one.

As an assessment of the limitations involved in applying these solutions, the respondents were asked which ones they considered as challenges and which ones as risks. Some of the limitations included the difficulties in technology and natural solutions, their implementation, maintenance, possible effects, and their adaptability in Panama City with the current policies. In total, it was found that from the 28 constraints, the participants voted 211 times for the challenge section with 59.94% of the total; on the other hand, the option of possible risk was selected 103 times, covering 29.26% of the constraints; at last, 10.80% of these were not considered a limitation or were not as relevant. Table 10 summarizes the results for this question, with the number of votes for each option.

**Figure 8:**
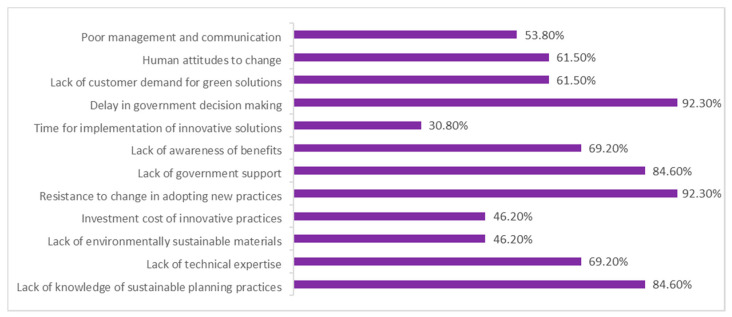
Factors that apply in the Panamanian context, according to respondents. Own elaboration.

As part of the research, respondents were asked what factors they considered that applied to Panama City. The most voted were the delay in government decision-making and resistance to change in the adoption of new practices, both with 92.30%. This was followed by the lack of government support and knowledge of sustainable planning practices with 84.60%. Figure 8 shows the results obtained:

Finally, they were asked for their opinions regarding this work, and they recommended or commented on the following factors:

- The viability of the options by approach is correct when considering the barriers in the Panamanian context, such as environmental awareness, user demand for green alternatives, and governmental will and support.
- To rely on bills that consider sustainable projects, such as Executive Decree No. 205 of 28 December 2000, which considers the approval of the Urban Development Plan for the Metropolitan Areas of the Pacific and Atlantic; as well as Executive Decree No. 139 of 1 September 2000, which considers the approval of special rules to maintain the character of a garden city in the interoceanic region.
- To consider the implementation of the architectural designs used in the decade of the 1960s, where the wind direction and open designs that allowed air circulation was contemplated.
- Keep in mind that the effects of climate change are experienced disproportionately by people with lower incomes. So take actions in vulnerable areas to natural disasters, including not applying designs that benefit or improve the quality of life only for the upper class or the more affluent.

## 5. Conclusions

The purpose of this work is the proposal of solutions using biomimicry in the context of Panama City. Through the weighting obtained in the Green City Index, three aspects were covered: energy, air quality and mobility, for which a biomimetic abstraction was obtained with the problem-based approach. During this analysis, 11 pinnacles were identified, and 17 solutions were proposed and described. Their concepts and applications were studied through an extensive literature review.

Biomimicry of ecosystems is sustained on a design criterion that is based on sustainable and regenerative principles; therefore, the support of professionals with this knowledge is important. Accordingly, a survey for experts in the relevant sectors was conducted. The results revealed that among the 17 proposed solutions, the most supported or feasible in the experts’ opinion were: arborization, green facades, solar roofs, e-mobility, green corridors, bicycle lanes, sidewalks and biofilters. In general, the concluding remarks from this paper and the final survey are following:

- Encouraging purification strategies can be achieved with the planning and use of natural adaptations: trees, green roofs/facades, green corridors and others, such as biofilters, titanium dioxide products, among others.
- Due to the heat island effect, it’s necessary to optimize the comfort of the inhabitants, considering the importance of trees in regulating the temperature of the soil and surrounding air. At the same time, the opportunities for heat regulation and energy savings presented by the application of biomimetic designs in buildings are recognized.
- The use of solar energy through photovoltaic systems was considered a vital pillar towards progress in the energy transition and distributed generation.
- The population must be aware of the impact of motorized transport and emissions caused by mobile sources for a modal shift that considers sustainable transport (bicycles, walking and electric alternatives).
- With the incorporation of greener infrastructure and solutions, government initiatives will be needed to create laws that encourage local photovoltaic systems, green roofs and facades, and other solutions in buildings; as well as e-mobility laws new projects for low-density transportation (bicycles, sidewalks).
- There is a need for collaboration with several professional bodies to ensure regulations to prevent the notorious pollution from the built environment.
- To achieve a regenerative city, fundamental changes and comprehensive strategies are needed in the form of long-term policies, rather than temporary compromises, as is the case for most political decision-making schemes in the country.

For future works, it is recommended to test the different proposals through simulations or new evaluations before implementation in this case study. Although the problem-based biomimetic analysis can be extended to other challenges that encompass the unassessed systems, such as how nature uses water, the methods presented should be revised before application to other case studies due to the specific challenges and factors identified for the pinnacles analysis and selection.

## Figures and Tables

**Table 1:** Summary of the evaluation for the Green City Index indicators (Ranges based on [19]).

No.	Category	Far below Average (0–20%)	Below Average (20–40%)	Average (40–60%)	Above Average (60–80%)	Well above Average (80–100%)
1	Energy and CO_2_			51.14%		
2	Land Use and Buildings			47.95%		
3	Transportation		39.00%			
4	Waste				69.08%	
5	Water				71.11%	
6	Sanitation				64.65%	
7	Air Quality	16.67%				
8	Environmental Governance				88.00%	
	Total Result			55.95%		

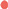
 Inadequate/poor, high risk, behind schedule. 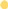 Reasonable, moderate risk, partially behind schedule 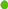 Good, low risk, on schedule.

**Table 2:** Comparison of Panama City and other Latin American cities from the Green City Index final evaluation. Adapted from [19].

Category	Far below Average (0–20%)	Below Average (20–40%)	Average (40–60%)	Above Average (60–80%)	Well above Average (80–100%)
Energy and CO_2_		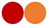	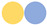	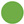	
Land Use and Buildings	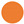	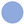	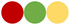		
Transportation		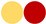	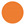	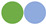	
Waste		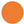		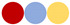	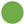
Water		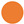	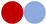	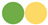	
Sanitation		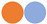	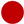	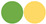	
Air Quality	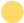	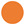	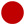	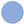	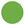
Environmental Governance		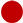	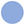	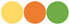	
Total Results		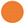	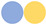		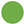
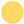 Panamá	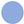 Quito	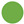 Curitiba	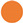 Montevideo	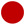 Porto Alegre	

**Table 3:** Strategies carried out by the selected pinnacles. Own elaboration.

Fenestraria aurantiaca	It can collect, filter and distribute light to photosynthetically active cells on the side of its body walls.	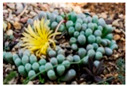	[21]
Photosyn-thesis	Plants use the chlorophyll pigment to absorb solar energy and donate electrons in the photosynthetic process, and to replace these electrons; they split the water molecule into hydrogen protons.	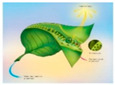	[22]
Termite mound	In their mounds, they implement the variation of wall thickness, the orientation of protruding structures and the application of an efficient design with air ducts that are close to the surface.	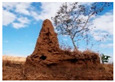	[23]
Elephant skin	The network of wrinkles on the surface of an elephant’s skin improves its thermoregulation by retaining water in the crevices along the skin.	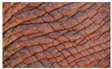	[24]
Trees and plants	Block sunlight and increase the surrounding humidity, resulting in a decrease in temperature, depending on characteristics, such as density, the thickness of foliage, leaf texture and clarity of color. Modulate the microclimate and sequester toxic compounds.	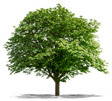	[25]
Strelitzia	It can contain elastic energy when an external force is applied to it, obtaining a reversible and repetitive mechanism, which can cover the sun from various angles.	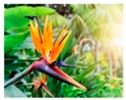	[26]
Hydroxyl radical	Controls the exposure time of certain organic compounds. It decomposes into water and oxygen and leaves no residual oxidants after biochemical reactions. Eliminates 99.9% of pathogenic microorganisms, destroys pollutant gases and reduces suspended particulate matter and COVs.	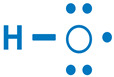	[27]
Saintpaulia	This plant has leaves that contain trichomes (tiny hairs) on their surface, thus trapping particles that can adhere to the leaf surface.	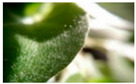	[28]
Microalgae	It can capture light and use its energy to absorb CO_2_ and other inorganic nutrients into its biomass. As a result of photosynthesis, they produce oxygen. In addition, they possess the ability to produce sugars for their structure and plant oils.	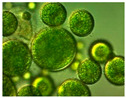	[29]
Physarum polyce-phalum	It can self-organize, spread out and form extensive and very efficient networks to find food sources. Moreover, it covers the shortest possible distances, making the best use of resources.	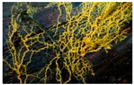	[30]
Ants colony	When they find a food source, they return to their nest, leaving behind a small amount of pheromone along the way. When other ants find this compound, they follow the trail. If they find food, they will reinforce the trail with more pheromones until they return to the colony. Therefore, there will be positive feedback that leads all ants to follow a single path.	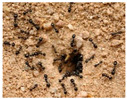	[31]

**Table 4:** Summary of proposed solutions for the implementation of cities. Own elaboration.

Solution	Description	Application in Other Parts of the World	Ref.
Solar roofs	It consists of photovoltaic solar energy as a primary or secondary source for buildings. The aim is to adapt the roofs of residential, commercial, and industrial buildings to house solar panels or collectors in 20–30% of the available space.	In countries like Vietnam, rooftop solar is booming. Despite the COVID-19 pandemic, Vietnam saw rooftop solar installations increase from having 378 MW in 2019 to 9583 GW, or a 2.43% increase from 2019. It now exceeds 100,000 systems in total.	[32]
Panels at bus stops and pedestrian walkways	Solar panels on bus stops as a reduction to the public grid energy system. The use of solar panels on pedestrian walkways in educational centers, government offices, shopping malls, parking lots, or public spaces, in general, is also being pursued.	They’ve been used as clean energy initiatives in China, Brazil, USA, India and more. For example, the Indian Institute of Technology, Kharagpur developed a successful 70m stretch of a pedestrian walkway with solar panels.	[33]
Solar sheets	Use of solar sheets, this innovation consists of triple laminated amorphous silicon glass. It can be used in those buildings that do not have enough space on the rooftops to install photovoltaic systems.	An implementation of solar sheets is seen in The Instituto Canario Superior de Estudios in Las Palmas de Gran Canaria, Spain. This building has a system of solar sheets on its façade, which function as windows.	[34]
Flectofin blinds	Adaptation of blinds in buildings, based on nature (bird of paradise flower). It works without hinges and with 90° displacements. It performs adaptive shading, efficiently covering buildings from solar radiation.	A Thematic Pavilion, which had been exhibited at Expo 2012 in Yeosu, Korea, was a kinematic façade that Soma architects designed. Individual kinematic fines have been applied in the façade for controlling daylight conditions.	[35]
Trees	Considers the use of trees and shrubs to improve the microclimate of urban areas through trees with dense foliage and light green, thick, rough leaves. In addition, by having a cool temperature, they will provide greater comfort while protecting from direct solar radiation.	In Taipei, Taiwan, the effects of twelve tree species were studied in subtropical urban areas. The most effective were *Ulmus parvifolia*, *Pterocarpu indicus* and *Ficus microcarpa*. In addition, the importance of leaf color, foliage density, leaf thickness and leaf surface roughness were concluded.	[36]
Sierpinski ceiling	The roof uses biomimicry to emulate the leaves of trees. The roof was made of fractals with a small exposure area that allows for better temperature distribution on the surface, thus obtaining lower temperatures. It is proposed for use in places, such as terraces, arbors, social or recreational areas, among others.	The National Museum of Emerging Science and Innovation (Miraikan) in Tokyo, Japan, built a fractal roof (Sierpinski forest) and compared the fractal prototype with a part of the roof made of PVC panels, concluding that the fractal surface had a much lower temperature.	[37]
Green hydrogen	The use of hydrogen as an alternative fuel in the country due to its zero-emissions benefit. In addition, it’s expected to establish a hydrogen distribution hub in Panama, according to its geographical position and the opportunities brought by the Panama Canal.	Hydrogen refueling stations currently exist in countries, such as Japan, the United States and Germany. H_2_ Energy Applications (in) Valley Environments (for) Northern Netherlands abbreviated HEAVENN.	[38]
Green walls and roofs	Implement green roofs/walls with native plants on buildings to cool the surrounding air through plant evapotranspiration. It converts any rooftop with more than 1300 square feet or at least 20% of the available space (for green or solar roofs) into green roofs. This would mean using natural barriers for CO_2_ reduction, divided into three types of roofs: intensive, semi-intensive and extensive.	In Cordoba, Argentina, a law was enacted in 2016, making it mandatory to convert any rooftop more significant than 1300 square feet, including new or existing, into green roofs.	[39]
Purifying plants	Use of trees and plants that trap volatile organic compounds through the opening and closing of leaf pores. This will contribute to the reduction of toxic pollutants in the city’s atmosphere and improve air quality.	In many places, mention is made of different species that could work as the most effective for sequestering emissions, such as VOCs, formaldehyde, benzene, carbon monoxide and trichloroethylene; these species are *Spathiphyllum*, areca palm, tiger tongue and *Chlorophytum comosum*.	[40]
Bio- filters	Implementation of microalgae-based biofilters in the busiest streets of the country, where traffic congestion levels are high and there is little space for planting large numbers of trees. Consideration is given to port areas where there are large logistical movements both at sea and land, industrial zones, etc.	There is the Biourban filter from Mexico. Its technicians assure that the models: BioUrban 2.0 and Bio Urban Industries, can supply the same amount of oxygen as 368 mature pine trees in a year, equivalent to the daily breathing of 2890 people. This filter has been incorporated in the Bus Terminal in Albrook Mall, in Panama City.	[41]
Photocatalytic cement	Composed of titanium dioxide, it is a coating used in avenues or sidewalks of public spaces and buildings with a large surface area exposed to sunlight. These can include constructing residential buildings, schools, bridges, hospitals and even monuments, especially in places with high pollution/odors.	In Milan, a 7000 m^2^ road surface was built with photocatalytic cement, obtaining a 60% reduction in the concentration of nitrogen oxide (NOx) at street level.	[42]
Walk to work, walk to school, walk to park	It involves strategies to mobilize citizens to their destinations, without vehicles (more emissions), by walking to their places of work, education, leisure or socio-cultural activities. Part of this strategy focuses on distributing these places in the city center, making less distance between the pedestrian’s home and its destination.	Lavasa Hill in India has a biomimetic design, which has land-use planning based on these concepts. Mobilization of residents through walking to their places of work, education, leisure or socio-cultural activities is implemented.	[43]
Sidewalks	Implementation of adequately designed and constructed roadways or sidewalks with permeable pavement and adequate space for pedestrians. Adequate and user-friendly measures, including the service strip (road signs, street lighting, street furniture, vegetation, among others).	Studies carried out on sidewalks in Cuenca, Barcelona, and Prague, consider the importance of using materials to construct sidewalks that can produce friction (cobblestones or concrete).	[44]
Green Corridors	It serves as a connection in the green areas of the urban zone, connecting different points of the metropolitan area using vegetation through its extension.	In cities, such as New York, Mexico City, Madrid, and Seoul, these corridors have been implemented along with flowers, trees, shrubs, walking paths, and bicycle lanes, thus improving the area’s average temperature.	[45]
Electric Transport	It covers the use of electric vehicles, including recharging points in different country areas, i.e., those near parking lots of shopping malls, supermarkets, offices, hotels, universities, etc. It would also include discouraging internal combustion vehicles and raising awareness of decarbonization issues at the national level.	In Europe, for example, the advancement of e-mobility was foreseen with ABB, which is the main technology partner and supplier of IONITY, a joint venture between different groups, such as BMW, Ford, Volkswagen, Audi and Porsche, whose goal was to operate a network of at least 400 fast charging points in 24 European countries by 2020.	[46]
Bicycle lanes	It involves creating a network of bikeway infrastructure, which aims for its realization in the city’s main roads and green areas. Its purpose is to serve as a connecting node to the rest of the city, which would increase the efficiency of mobility in the urban area in a sustainable and emission-free manner.	The countries with the most extended distances covered by cycling have been studied, determining this activity as the main form of urban mobility. It was concluded that the countries with the longest distances (600 km to 900 km) are: Netherlands, Denmark, and Belgium. Furthermore, in Germany, between 1994 and 2017, distance cycled per capita increased by over 150 km, consistent with an increase of over 50%.	[47]
Routing algorithm	Use of algorithms based on nature, focused on minimum use of resources and high efficiency, resulting helpful in the creation of future networks of the Panama Metro, by optimizing the city’s roads. Its use is considered in those logistic services existing in Panama City.	In the cities of La Paz and El Alto in Bolivia, modeling work was carried out for the combinatorial optimization of transportation flow patterns by applying the ant colony algorithm and Dijkstra’s algorithm (minimum paths algorithm).	[48]

**Table 5:** Summary of methodological sheets of indicators based on Ecosystem Services Analysis. Own elaboration.

Indicator Name	Brief Description	Input Variables and Their Respective Unit	Indicator Calculation Formula	Indicator Evaluation (IR):	Data Source
Habitat provision	It shows the calculation of the number of existing trees in the land area of the townships of Bella Vista, Betania, Calidonia, San Felipe, San Francisco and Santa Ana.	-Number of tree units (NUA): Unit	IPH = NUA STT	NUA = 22,334 trees	Municipality of Panama and National Institute of Statistics and Census [50].
		-Total territorial surface (STT): km^2^		STT = 12.3 km^2^	
		-Habitat Provision Indicator (IPH): Trees/km^2^		IPH = 1815.77 trees/km^2^	
Nutrient cycling	It shows the ratio of tons of recycled garbage (BR) to waste disposed of in landfills (BT) for the capital city.	-Recycled waste (BR): tons per year (ton/year)	IR = BR × 100 BT	BR= 11,700 ton/year	Urban and Household Cleaning Authority of Panama [51]
		-Total waste disposed of (BT): tons per year (ton/year)		BT= 583,576.60 ton/year	
		-Recycling indicator (IR): Percent (%)		IR = 2.00%	
Climate regulation	Measurement of the amount of emissions absorbed (TCO_2_e) by hectares of forest without change of use (HB) of the metropolitan area under study.	-Hectares of forested area in the study site (HB): hectare	Iabs = tCO_2_e HB	CO_2_e = 948,863 tCO_2_e/year	IDOM, Municipality of Panama [52].
		-Tons of CO_2_e absorbed per year (CO_2_e): tCO_2_e/year		HB = 178,850 ha	
		-Emission absorption indicator (Iabs): tons CO_2_e/hectare-year		Iabs= 5.31 tCO_2_e/hectare	
Purification of air	Measuring the amount of green areas in the city of Panama (AV) over the total urban area (STU) excludes forests, mangroves, bodies of water, and agricultural land.	-Hectares of green areas in the city (AV): hectare	IP = AV × 100 STU+AV	AV = 360.60 ha	Panama City Hall, IDOM [53].
		-Total hectares of the urban area (STU): hectare		STU = 36,928 ha	
		-Air purification indicator (IP): Percent (%)		IP = 0.97%	
Provision of freshwater	It shows the amount of rainwater used in buildings in residential areas, economic centers, shopping centers, and other urbanizations.	-Precipitation in Panama City per year (PCP): L/m^2^	VT = PCP × AT	PCP = 1850.20 L/m^2^	Regional Water Resources Committee, Panama City Hall, IDOM [52,54].
		-Hectares of residential and non-residential areas (AT): hectare		AT =271,584,000 m^2^	
		-Indicator of rainwater volume per year in the study area (VT): L/year		VT = 517,965 millions of liters	
Provision of energy	Shows the calculation of the amount of renewable energy consumed compared to the total energy consumed in the province of Panama.	-Renewable energy consumed in the province of Panama (ERC): MWh	IER= ERC × 100 ETC	ERC = 391,381.8 MWh	National Public Utilities Authority (ASEP) [55,56].
		-Total energy consumed in the province of Panama (ETC): MWh		ETC = 3,266,682.7 MWh	
		-Renewable Energy Indicator (IER): Percent(%)		IER = 11.98%	

**Table 6:** SWOT analysis for energy initiatives.

SWOT Analysis	Strengths	Weaknesses
Internal	**Solar panels:** No emissions. It makes use of the available space in buildings, residences, etc. The sun is a usable and unlimited source of energy. **Panels with microalgae:** Uses the sun and CO_2_ to produce biomass. Microalgae purify the air at a higher rate than trees [59]. **Renewable hydrogen:** Emission-free consumption and high calorific value, can be mixed with other gases, serves as electricity storage (green hydrogen). It can be obtained in different forms (blue, gray and green). **Sierpinski roof:** It allows a better temperature distribution on the surface due to its fractals with a small exposure area; lower temperatures are obtained [37]. **Flectofin louvers:** Operates without hinges and with 90° displacements. It performs an adaptive shading, efficiently covering the buildings. **Green facades:** It represents a natural barrier for the buildings against solar radiation and with the evapotranspiration of the leaves the surrounding temperature is reduced.	**Solar panels:** Requires an initial investment, needs direct solar radiation and no permanent shade. They occupy considerable space. **Panels with microalgae:** Requires solar radiation and equipment suitable for transforming it into electricity [59]. **Renewable hydrogen:** Probability of leakage, volatile, needs a lot of logistical and storage care. Green requires high renewable energy generation and is expensive to produce (electrolysis).Sierpinski roof: Partially transmits sunlight [37]. **Flectofin blinds:** They do not have automatic control, so they would have to be adapted. **Green facades:** Depending on the type of green roof, it can generate a lot of extra weight. Some types of green roofs or walls require maintenance, watering, and pruning.
	**Opportunities**	**Threats**
External	**Solar panels:** This would increase the distributed generation market in the region, contributing to the decarbonization of the energy matrix. Energy is in trend, so the cost decreases over time, making it more feasible. Therefore, energy savings are obtained. **Microalgae panels:** Can use the remaining biomass from the process to generate biogas. **Renewable hydrogen:** Growing global market, Panama’s advantageous position in distribution. **Sierpinski roof:** Can be adapted to limited sites for planting trees, including terraces, pergolas, social or recreational areas, among others. **Flectofin blinds:** Studies and prototypes based on biomimicry are growing globally. **Green facades:** Diminish the heat island effect in the urban environment and purify the air. They can reduce rainwater reaching the street level and its flooding.	**Solar panels:** Buildings and trees generate shadows. Low performance in rainy or cloudy weather. **Renewable hydrogen:** There is no gas distribution network in the country. Lack of sufficient demand in industries. **Microalgae panels:** Shadows may be cast by buildings adjacent to the installation. Technology under development. **Sierpinski roof:** There is no record of its application in Panama. Designers and citizens do not know it. **Flectofin blinds:** There is competition in the local market, where biomimicry is unimportant for users. **Green facades:** Poor selection or maintenance of the species can lead to a deterioration of the roof or wall, in addition to dangers due to biological diseases and the climate present in the site.

**Table 7:** SWOT analysis for the proposed air quality initiatives.

SWOTAnalysis	Strengths	Weaknesses
Internal	**Trees:** Minimize air and noise pollution, sequester harmful compounds for living beings. **Green facades:** Filter pollutants and heavy metals from rainwater, grow fruits, flowers, or vegetables, atmospheric filter pollutants, such as CO_2_ and act as an acoustic barrier. **Biourban microalgae filter:** They can sequester up to 2 tons of CO_2_ per year and pollutants, such as CO and NOx. Their oxygen generation capacity is equivalent to that of hundreds of trees [60]. **Photocatalytic concrete:** Use of photons to neutralize organic and inorganic pollutants. It makes surfaces self-cleaning. Savings in maintenance costs due to their durable nature [42].	**Trees:** They require maintenance and inventory of their phytosanitary status. In addition, some have to rot in the heart of the tree trunk [61]. **Biourban microalgae filter:** The indoor model requires an investment due to its energy consumption. All models require maintenance every 3 to 6 months [60]. **Photocatalytic concrete:** For best performance, surfaces should preferably be exposed to direct sunlight [42].
	**Opportunities**	**Threats**
External	**Trees:** Plans have been presented that address their implementation in the city. There is a growing awareness among the population about tree planting and its benefits [61]. **Biourban microalgae filter:** There are various models for different sites, such as industrial, indoor, outdoor and ashtrays. They can be installed on the main roads most susceptible to vehicular congestion in the city. Other places to be considered are port areas, logistics centers and industrial areas [60]. **Photocatalytic concrete:** The construction sector can adapt these technologies to trends, such as bridges, office or residential buildings, hospitals, monuments, schools and drainage structures [42].	**Trees:** They can affect road infrastructure, power lines, buildings, and their roots can cause sidewalks or walls to rise. Lack of trees in some regions of the city [61]. **Biourban microalgae filter:** In indoor locations, it is necessary to consider the space required due to their size. In addition, they can weigh from 120 kg to 1 ton [60]. **Photocatalytic concrete:** There are several products for conventional concrete that are manufactured locally. Cement plants in the region do not produce this type of cement.

**Table 8:** SWOT analysis for the proposed mobility initiatives.

SWOTAnalysis	Strengths	Weaknesses
Internal	**Citizen mobilization strategies (sidewalks, walk to work, routing algorithm, bicycle lanes):** Greater efficiency in terms of citizen mobility. Properly constructed sidewalks promote walking and improve connectivity. Walking and bicycling will increase people’s physical fitness. They consider the frequency of use and the space needed for different people, including those with reduced mobility, blind people, etc. They provide safe walking with their materials and add permeability to the routes [43]. **Green corridors:** Increase biodiversity, connect, and give continuity to green spaces. Can serve as a site for recreation and leisure, as they facilitate walking and cycling through bicycle paths. It reduces air pollution in cities [62]. **E-mobility:** Generates zero emissions, has lower noise levels, low maintenance due to fewer mechanical parts, and is lighter. The energy efficiency of the engine is higher than that of conventional combustion engines [63].	**Citizen mobilization strategies (sidewalks, walk to work, route algorithms, bicycle lanes):** The user prioritizes his comfort due to the city’s changing weather, which prevents him from transitioning from his vehicle to walking.Not all people have the condition and motor capacity for long walks or frequent use of bicycles. The latter requires good care and maintenance [43]. **Green corridors:** Constant maintenance, pruning, fertilization, and irrigation are needed to strengthen the ecosystems where insects, mammals, and birds have arrived. Diseases can appear in selected tree species. **E-mobility:** Electric car technology is more expensive than a combustion engine car. Its autonomy is limited, serving only to move around the city and its surroundings. In addition, it requires a recharging point in the garage, where recharging can be slower [63].
	**Opportunities**	**Threats**
External	**Citizen mobilization strategies (sidewalks, walk to work, route algorithms, bicycle lanes):** They reduce the use of vehicles and optimize routes, i.e., they will contribute to fewer traffic jams, which will result in fewer polluting emissions. Permeable sidewalks minimize the frequency of flooding. Better sidewalks can accommodate more space for street furniture (stops, benches, trash cans) or necessary vegetation. Walking or bicycling would save on fuel use and encourage people to become aware of their emissions. Bicycles are trending worldwide, and cyclists are less likely to suffer from cardiovascular diseases [43]. **Green corridors:** They would allow an improvement of the urban synergy between nature and society. It can be used as a strategy to avoid heat island effects in small cities. It would take less time to walk between city squares and parks, thanks to the comfort it provides to pedestrians [62]. **E-mobility:** Panama is in the process of transition through the 2020–2030 energy plan. If implemented, incentives for the technology could increase in the trend, resulting in a considerable decrease in emissions [63].	**Citizen mobilization strategies (sidewalks, walk to work, routing algorithms, bicycle lanes):** The design of the city structure still represents a challenge to implement more efficient routes in the metropolitan area. It’s not feasible to apply some or all of the strategies in certain parts of the city due to its distribution’s lack of initial planning. Priority is given to vehicular space, limiting the pedestrian area. Weather is an aspect that can influence walking and cycling strategies. Sidewalks’ condition (low friction/raised sidewalks) can result in pedestrian injuries, reduced walking performance, or accidents. Bicycling has a risk in cities with few bicycle lanes and a high flow of vehicles. Accidents can occur due to a lack of protective equipment, signaling, or irresponsible drivers [43]. **Green corridors:** It is challenging to implement green spaces due to the complexity of the original design of Panama City, where priority is given to vehicular spaces [62]. **E-mobility:** Public charging infrastructure is very low-Lack of current government incentives and trained technical professionals in the country. Due to its manufacture and the electricity consumption from conventional energy sources [63].

**Table 9:** Feasibility of nature-based solutions for Panama City: expert opinion. Own elaboration.

Solutions	5	4	3	2	1	Possibility Score (1–5)	Rk (Rank)
Solar roofs	53.85%	23.08%	7.69%	15.38%	0.00%	4.15	4
Panels at bus stops/pedestrian walkways	38.46%	23.08%	15.38%	23.08%	7.69%	3.85	7
Solar sheets	23.08%	46.15%	15.38%	15.38%	0.00%	3.77	8
Flectofin blinds	7.69%	30.77%	53.85%	7.69%	0.00%	3.38	11
Trees	69.23%	23.08%	7.69%	0.00%	0.00%	4.62	2
Sierpinski ceiling	23.08%	23.08%	30.77%	15.38%	0.00%	3.31	12
Renewable hydrogen	30.77%	23.08%	7.69%	38.46%	0.00%	3.46	10
Green walls and roofs	38.46%	46.15%	15.38%	0.00%	0.00%	4.23	3
Purifying plants	76.92%	23.08%	0.00%	0.00%	0.00%	4.77	1
Biofilters	23.08%	53.85%	23.08%	0.00%	0.00%	4.00	6
Photocatalytic cement	23.08%	23.08%	38.46%	7.69%	7.69%	3.46	10
Walk to work, walk to school, walk to the park	30.77%	15.38%	30.77%	15.38%	7.69%	3.46	10
Sidewalks	46.15%	23.08%	23.08%	7.69%	0.00%	4.08	5
Green corridors	30.77%	46.15%	23.08%	0.00%	0.00%	4.08	5
Electric transport	46.15%	38.46%	7.69%	7.69%	0.00%	4.23	3
Bicycle lanes	38.46%	46.15%	7.69%	7.69%	0.00%	4.15	4
Routing algorithm	23.08%	46.15%	15.38%	7.69%	0.00%	3.62	9

**Table 10:** Limitations of the nature-based solutions for Panama City. Own elaboration.

Limitations	Challenge	Risk	None
Low panel performance on rainy or shaded days.	4	7	1
Solar panels require a high initial investment.	7	3	3
Microalgae panels require equipment suitable for biomass-to-electricity conversion.	7	4	1
Renewable H_2_ production is very costly (electrolysis).	6	6	0
Hydrogen is volatile and has a high probability of leakage.	2	10	0
There is no gas distribution network in the country.	10	2	0
In the application of a Sierpinski roof and/or Flectofin louver, biomimicry is not so important yet for Panamanian users.	10	0	3
Trees require maintenance and inventory of their phytosanitary condition.	8	0	5
Trees can affect road infrastructure, power lines and cause sidewalks and walls to rise.	3	8	2
Lack of tree planting in areas of the city.	11	1	1
The Biourban filter requires an investment due to its energy consumption.	7	4	0
Filters require maintenance every 3 to 6 months.	6	5	1
Consider the space to implement the filter due to its size.	6	3	2
Photocatalytic concrete needs direct sunlight preferably.	3	4	5
Local cement plants do not produce this type of product (with titanium dioxide).	8	3	2
The user prioritizes his comfort when moving around.	7	2	3
The climate prevents the transition from using a vehicle to walking or cycling.	10	2	1
The current design of the city is inefficient.	9	4	0
Priority is given to vehicular space over pedestrian space.	10	3	0
Lack of land-use planning and urban distribution.	8	5	0
Sidewalks are in poor condition (raised, low friction).	8	5	0
The current signage of bicycle lanes, and the danger for possible accidents with cars.	7	5	1
Constant maintenance of green corridors.	11	1	1
Difficulty in implementing green spaces due to city design.	10	3	0
Technology for electric mobility is more expensive than traditional technology.	9	4	0
Limited autonomy (few electric charging points).	8	4	1
Requirement for government incentives.	10	1	2
Emissions still exist during electric charging if it comes from conventional sources.	6	4	3
Total results	211	103	38

## Data Availability

Not Applicable.

## Appendix A

**Table A1:** Summary of the evaluation of indicators in the Green City Index. Adapted from [19].

Category	Indicator	Type	Data	Source	Weight	Standard	Calculation	Result
Energy and CO_2_	CO_2_ emissions from per capita electricity consumption	Quantitative	284.93 kg/person	[17]	25%	202.20 kg/average person	17.81	0.1781
	Electricity consumption per unit of GDP or per capita	Quantitative	2226 kWh/person	[64]	25%	<815 KWh/person	−43.28	0.00%
	Clean energy policy.	Qualitative	Official Gazette (Law 37 of 2013, 45 of 2004, 44 and 42 of 2011).	[4,17,65,66]	25%	0–3	2.00	16.67%
			National Energy Plan 2015–2050					
			2nd Biennial Report					
	Climate Change Action Plan.	Qualitative	URRE Law	[64]	25%	0–3	2.00	16.67%
			National Energy Plan 2015–2050					
			Paris Agreement					
			Montreal Protocol					
			2nd Biennial Report					
Land use and Buildings	Green spaces per capita	Quantitative	8 m^2^/inhabitant	[53]	25%	100 m^2^/hab	2.00	2.00%
	Population density.	Quantitative	5867 hab/km^2^	[53]	25%	<7000 hab/km^2^	20.95	20.95%
	Green building policy.	Qualitative	Sustainable Building Guide	[67]	25%	0–3	2.00	16.67%
			Sustainable Building Regulations					
	Land use policy.	Qualitative	National Land Management Policy	[61,68]	25%	0–3	1.00	8.33%
			National Forestry Strategy, REDD					
			Forestry Incentives Law of 2017					
			Arborization Plan					
Transportation	Length of the public transport network.	Quantitative	0.03 km/km^2^	[53]	25%	>0.3 km/km^2^	−1.01	0.00%
						<7 km/km^2^		
	Stock of cars and motorcycles.	Quantitative	0.22 vehicles/hab	[53]	25%	0.3 vehicles/hab	14.00	14.00%
	Urban public transport policy.	Qualitative	Comprehensive Sector Program	[68]	25%	0–3	2.00	16.67%
			Integral Urban Mobility Plan					
	Congestion reduction policy.	Qualitative	Integral Plan for the Improvement of Mobility and Road Safety	[69,70]	25%	0–3	1.00	8.33%
			Panama Subway					
			ATTT, MOP, MUPA					
Waste	Proportion of waste collected and disposed properly.	Quantitative	92%	[53]	25%	>70%	32.86	25.00%
	Waste generated per capita.	Quantitative	456.25 kg/hab/year	[71]	25%	800 kg per person	10.74	10.74%
	Waste collection and disposal policy.	Qualitative	National Waste Management Plan 2027	[53,72,73]	25%	0–3	2.00	16.67%
			Plan for Service Improvement (AAUD)					
			Panama City Municipal Integrated Waste Management Plan					
	Waste recycling and reuse policy.	Qualitative	Zero Waste Plan	[72]	25%	0–3	2.00	16.67%
			Cleaner Production Policy					
Water	Per capita water consumption	Quantitative	274 L/person/day	[53]	20%	Min: 60 lt/day	−43.98	0.00%
						Max: 126.9 lt/day		
	Water leaks	Quantitative	40%	[73]	20%	<45%	17.78	17.78%
	Population with access to potable water.	Quantitative	96%	[53]	20%	>80%	24.00	20.00%
	Water quality policy.	Qualitative	National Water Security Plan 2015–2050	[74,75]	20%	0–3	3.00	20.00%
			IDAAN Action Plan 2019–2024					
	Water sustainability policy.	Qualitative	National Water Security Plan2015–2050	[53,74,75,76,77]	20%	0–3	2.00	13.33%
			Metropolitan Panama Action Plan					
			IDAAN Action Plan 2019–2024					
			Government Strategic Plan 2020–2024					
Sanitation	Population with access to improved sanitation.	Quantitative	68%	[53]	33%	93.70%	23.95	23.95%
	Proportion of wastewater treated.	Quantitative	31%	[53]	33%	Min: 10%	7.70	7.70%
						Max: 100%		
	Sanitation policy.	Qualitative	Metropolitan Panama Action Plan	[53,78]	33%	0–3	3.00	33.00%
			Panama Sanitation Program					
Air quality	Nitrogen dioxide concentration levels.	Quantitative	36 μg/m^3^ per day	[51]	25%	≤20 μg/Nm^3^ per day	−20.00	0.00%
	Sulfur dioxide concentration levels.	Quantitative	-	-	25%	-	0.00	0.00%
	Concentration levels of suspended particulate matter.	Quantitative	49 μg/m^3^ per day	[53]	25%	≤20 μg/Nm^3^ per day	−24.00	0.00%
	Clean air policy.	Qualitative	Executive Decree No. 5 of 4 February 2009.	[53,79,80,81]	25%	0–3	2.00	16.67%
			Executive Decree No. 38 of 3rd June 2009.					
			Technical Regulations DGNTI-COPANIT 43-2001.					
			Panama Metropolitan Action Plan					
Environmental Governance	Environmental management.	Qualitative	National Institute of Statistics and Census	[15,50,81]	33%	0–3	3.00	33.00%
			The National Climate Change Strategy 2050					
			Municipality of Panama					
			Committee Against Climate Change					
			2nd Biennial Report					
			1st Nationally Determined Contribution					
	Environmental monitoring.	Qualitative	National Institute of Statistics and Census	[15,18,50,81]	33%	0–3	3.00	33.00%
			National Climate Change Strategy					
			Water Quality Monitoring (MiAmbiente, Ministry of Health and IDAAN)					
			Panama’s Urban and Household Sanitation Authority					
			Air Quality Monitoring by the ACP					
	Citizen participation.	Qualitative	Ministry of Environment	-	33%	0–3	2.00	22.00%
			National Assembly					
			National Government					
